# Non-cross-linked biological mesh in complex abdominal wall hernia: a cohort study

**DOI:** 10.1007/s00423-020-01881-4

**Published:** 2020-04-22

**Authors:** Ruth Kaufmann, Friedrich-Eckart Isemer, Christoph W. Strey, Johannes Jeekel, Johan F. Lange, Guido Woeste

**Affiliations:** 1 Department of Surgery, Erasmus University Medical Center, Room Ee-173, Wytemaweg 80, NL-3015 CN Rotterdam, The Netherlands; 2 Department of Radiology, Haga Teaching Hospital, The Hague, The Netherlands; 3 Department of General and Trauma Surgery, St. Josefs-Hospital, Wiesbaden, Germany; 4 Hernienzentrum DKD HELIOS-Klinik, Wiesbaden, Germany; 5 Department of General, Visceral, and Vascular Surgery, Diakoniekrankenhaus Friederikenstift gGmbH, Hannover, Germany; 6 Department of Neuroscience, Erasmus University Medical Center, Rotterdam, The Netherlands; 7 Department of General and Visceral Surgery, Klinikum der Johann Wolfgang Goethe Universität Frankfurt, Frankfurt am Main, Germany; 8 Department of General and Visceral Surgery, Agaplesion Elisabethenstift gGmbH, Darmstadt, Germany

**Keywords:** Complex abdominal wall hernia, Non-cross-linked biological mesh, Long-term results

## Abstract

**Purpose:**

Complex abdominal wall hernia repair (CAWHR) is a challenging procedure. Mesh prosthesis is indicated, but the use of synthetic mesh in a contaminated area may add to overall morbidity. Biological meshes may provide a solution, but little is known about long-term results. The aim of our study was to evaluate clinical efficacy and patient satisfaction following Strattice™ (PADM) placement.

**Methods:**

In this cohort study, all patients operated for CAWHR with PADM in three large community hospitals in Germany were included. Patients underwent abdominal examination, an ultrasound was performed, and patients completed quality-of-life questionnaires. The study was registered in ClinicalTrials.gov under Identifier NCT02168231.

**Results:**

Twenty-seven patients were assessed (14 male, age 67.5 years, follow-up 42.4 months). The most frequent postoperative complication was wound infection (39.1%). In no case, the PADM had to be removed. Four patients had passed away. During outpatient clinic visit, six out of 23 patients (26.1%) had a recurrence of hernia, one patient had undergone reoperation. Five patients (21.7%) had bulging of the abdominal wall. Quality-of-life questionnaires revealed that patients judged their scar with a median 3.5 out of 10 points (0 = best) and judged their restrictions during daily activities with a median of 0 out of 10.0 (0 = no restriction).

**Conclusions:**

Despite a high rate of wound infection, no biological mesh had to be removed. In some cases, therefore, the biological meshes provided a safe way out of desperate clinical situations. Both the recurrence rate and the amount of bulging are high (failure rate 47.8%). The reported quality of life is good after repair of these complex hernias.

## Introduction

Incisional hernia is a common complication after abdominal surgery. Incidences range from 3 to 20% in the general population [1–7] with an increased incidence of 26 up to 39% in patients suffering of obesity or aortic aneurysms [2, 7–19]. Currently, incisional hernias are most often reinforced with mesh material [20, 21]. The use of mesh radically lowered the 10-year recurrence rates after incisional hernia repair [20]. There are various mesh prosthesis available (conventional synthetic meshes, biological meshes, and since recently also biosynthetic meshes (i.e., slowly resorbable synthetic meshes)) [22–24]. Conventional synthetic meshes are still used most often in general practice, and polypropylene mesh is the most popular product [25].

There are various reasons, like mesh infections, enterocutaneous fistulas, burst abdomen, and anastomotic leakage, that could turn an ‘uncomplicated’ ventral hernia repair into a complex abdominal wall hernia repair (CAWHR) [26].

The grade of contamination is an important factor in the treatment of incisional hernia. To classify the amount of contamination, the Ventral Hernia Working Group (VHWG) developed a hernia grading system to classify the different grades of contamination and its treatment complexity in abdominal wall hernia repair (grade 1: low risk to grade 4: infected) [27]. In potentially contaminated and infected incisional hernia the use of conventional synthetic mesh is controversial and might lead to a higher morbidity and even mortality. In case of an infected synthetic mesh, the mesh should be removed completely to achieve complete wound healing [28]. Patients often have multiple comorbidities and risk factors that increase the risk for postoperative complications and hernia recurrence. In order to provide the safest individual treatment strategies to high morbidity patients, a classification for complex abdominal wall hernias was defined to facilitate the choice for CAWHR concepts [29].

Patients who need CAWHR often undergo strictly planned, staged repair of their hernia defect [30]. Part of a different treatment strategy for CAWHR is the use of biological mesh instead of synthetic mesh in a one-stage procedure. Biological mesh is however only seldom used, since costs per prosthesis are high and little is known about long-term results. In this study, the use of Strattice™ mesh was evaluated. Strattice™ is a decellularized, intact, non-cross-linked porcine acellular dermal matrix, derived from porcine dermis [31]. The study is initiated after a nationwide German questionnaire of the ROKI Group [32]. The aim of this study was to evaluate clinical efficacy and patient satisfaction following Strattice™ placement in patients treated for CAWHR in three academic and peripheral hospitals in Germany. In this study, Strattice™ is referred to as non-cross-linked porcine acellular dermal matrix (PADM).

## Material and methods

### Study design

A cohort study was performed in three large community hospitals in Germany (both academic and peripheral centers). Patients were identified retrospectively and were invited to an outpatient clinic appointment. Ethical approval for this study was obtained from the Ethics Board of the Johann Wolfgang Goethe University Frankfurt, Germany. After ethical approval from the Johann Wolfgang Goethe University Frankfurt, ethical approval was obtained from all local ethical committees in the participating hospitals.

### Patients

Patients were eligible for inclusion in the cohort if they had been operated with PADM for the indication CAWHR in one of the participating centers in Germany. CAWHR was defined as the repair for a potentially contaminated to infected hernia, which is grade 3 to grade 4 according to the system developed by the VHWG [27]. Patients had to provide written informed consent to participate in the study.

### Procedure

Patients were identified by the surgeons of the participating centers. The surgeons contacted all their patients and gave them—if they were interested—additional information about participation in the study. After written informed consent was obtained, patients were invited to an appointment in the outpatient clinic.

Patients were interviewed to collect baseline parameters, and to assess their medical history. Baseline parameters were defined as age, gender, BMI, severity of co-morbidity score [33], length of follow-up, smoking history, and occupational heavy lifting. The assessment of their medical history focused on medical conditions like COPD/chronic coughing, steroid use, malignancy, diabetes, general abdominal operations, and specific abdominal wall operations.

All patients underwent abdominal wall examination to assess the presence of a hernia recurrence or bulging of the abdominal wall. An abdominal wall ultrasound was performed in case of doubt. Patients completed quality-of-life questionnaires to assess patient satisfaction. The EuraHS quality-of-life questionnaires were used to assess patients’ quality of life [33, 34]. The STROBE statement was followed [35].

The acquired data was registered in the standardized case record forms of the incisional ventral hernia route in the EuraHS Database (http://www.eurahs.eu/). These case record forms were registered in a private group and were only accessible to the members of this study group.

### Outcomes

The primary outcome for this study was recurrence of hernia and / or the bulging of the abdominal wall after PADM repair. Both incisional hernia and the recurrence of incisional hernia were defined as any abdominal wall gap with or without bulge in the area of a postoperative scar perceptible or palpable by clinical examination or medical imaging [36]. Bulging of the abdominal wall was defined as a substantial increase in abdominal circumference, not explicable by weight gain, in the absence of a palpable or objectifiable fascia defect, and observed by either the patient or the doctor [37–39]. All patients were diagnosed by clinical examination. In case of doubt, abdominal wall medical imaging (i.e., ultrasound and/or CT scan) was performed to confirm or reject the diagnosis.

Secondary outcomes in this study included postoperative complications, i.e., wound infection, mesh infection, intra-abdominal and/or skin abscess, seroma, hematoma, necrotic abdominal wall, fistulas, and burst abdomen. The occurrence of mesh explantation was also recorded, just like additional abdominal operations, and quality-of-life parameters. Complications were classified according to Dindo and Clavien [40].

### Statistical analysis

Continuous variables were summarized by using means and ranges; categorical values were summarized with frequencies and percentages. Quality-of-life questionnaires were summarized with medians and ranges. The database was made in Microsoft Excel version 2016 (MSO 16.0.11029.20045) and analyzed in SPSS version 26.0 (version 26.0.0.0). The data was assessed for normality by performing a Shapiro-Wilk’s test (*p* > 0.05), and a visual inspection of their histograms, normal Q-Q plots, and box plots showed that most of the data was approximately normally distributed. Thereafter, the skewness and kurtosis of the data were calculated. If the Z-values were in the desired range of − 1.96 to 1.96, the data was presented as median (range). If the data was not normally distributed, it was presented as median (interquartile ranges). There was no data safety committee overseeing the study. The study was registered in ClinicalTrials.gov under Identifier NCT02168231.

### Role of the funding source

Funding to execute this study was obtained from LifeCell Corporation, a KCI company, Branchburg, NJ, USA. The funder of the study had no role in study design, data collection, data analysis, data interpretation, or writing of the report. The corresponding author had full access to all the data in the study and had final responsibility for the decision to submit for publication.

## Results

### Patients characteristics

A total of 27 patients met the inclusion criteria, of whom four were deceased. These four patients had passed away after surgery after 5, 22, 50, and 904 days, respectively. Twenty-three patients have been assessed for long-term follow-up (14 male, mean age 67.5 years, mean follow-up 42.4 months). Their median BMI was 27.4 (interquartile range 24.1–32.2). The median SOC score was 2 for all registered comorbidities (Table 1).

**Table 1:** Baseline criteria

Mean follow-up (mean)	42.4 months
Gender: male vs. female	14 vs. 9
Age (mean) (range)	67.5 years (48–90)
BMI (median) (interquartile range)	27.4 (24.1–32.2)
SOC score (median) (range)	2 (1–3)
Comorbidities (number of patients (percentage))	
Arterial hypertension	9 (39.1%)
Cardiac disease	6 (26.1%)
Diabetes mellitus type II	8 (34.8%)
Malignant disease	2 (8.7%)
Pulmonary disease (COPD/asthma)	3 (13%)
Renal disease	5 (21.7%)
Other: peritonitis, fistula, cachexia, hypothyroidism, portal vein thrombosis	8 (34.8%)
No comorbidities	5 (21.7%)
Smoking (daily/occasional smoking)	6 (26.1%)
Ex-smoker	7 (30.4%)

### Perioperative information

The risk factor seen most often was a personal history of previous abdominal wall hernia operation (Table 2). The estimated diameter of the hernia was median 18.25 cm (range 10–30 cm). The median defect size of the hernia was 357 cm^2^ (range 100–900 cm^2^). The operation was elective in 16 patients, and an emergency procedure in five patients. In two patients, it was unknown whether it was an elective or an emergency procedure. All perioperative information was recorded retrospectively and whenever possible verified with patients during their outpatient visit.

**Table 2:** Risk factors for complex abdominal wall hernia (number of patients (percentage))

Abdominal aortic aneurysm	1 (4.3%)
Anticoagulation therapy	2 (8.7%)
Chronic use of cortisone	1 (4.3%)
No other risk factors	7 (30.4%)
Personal history of previous abdominal wall hernia operation	9 (39.1%)

The hernia was located most often in the midline in the areas M2, M3, M4, and M5. Only three patients had undergone a previous hernia repair. These patients had undergone a median number of two previous hernia repairs (range one to three operations). All patients were operated under general anesthesia. Most patients received a single dose of antibiotics preoperatively (*n* = 11). In none of the patients, preoperative botulinum toxin or progressive preoperative pneumoperitoneum was used.

The wound classification was median CDC wound class III (contaminated) [41, 42]. In ten patients, a part of the bowel had to be removed during operation. The abdomen was closed with a combined component separation and mesh placement in 15 patients. In eight patients, mesh repair sufficed. All patients received a Strattice™ mesh. The most frequently used mesh size was 400 cm^2^. The mesh was fixated with transfascial sutures, and the entire hernia defect could be closed in all patients. There were no intraoperative surgical complications. The median length of stay after operation was 15 days (range 7–124 days).

### Postoperative outcomes

The most frequent postoperative complication was wound infection (39.1%). In six patients, it was a superficial wound infection. In no case, the PADM had to be removed. Other intrahospital complications were a bleeding complication in one patient, and in three patients there were general complications (acute renal failure, pleural effusion, peritonitis). In five patients, there were no intrahospital complications. The classification of the complications according to Dindo and Clavien was median grade IIIb (intervention under general anesthesia) [40].

### Long-term outcomes

By the time of outpatient clinic visit, six out of 23 patients (26.1%) had a recurrence of hernia, of whom one patient had undergone reoperation. All recurrences were found at the original hernia site. Another five patients (21.7%) had an asymptomatic bulging of the abdominal wall. Fourteen patients (60.1%) were evaluated by clinical examination combined with medical imaging, seven patients (30.4%) were evaluated with clinical examination, and in two patients (8.7%), it was unknown whether clinical examination was used with or without medical imaging. Patients with a recurrence or bulging often wore an abdominal binder.

### Quality-of-life parameters

Patients reported a good quality of life on the EuraHS quality-of-life questionnaires (Table 3). The quality-of-life data are presented as median scores. Patients had no pain in rest or during activities, and the worst pain they had felt in the last week was no pain, although one patient (4.3%) reported a pain score of 10. Most patients had no restriction in their daily activities. They experienced only minor limitations in their activities outside the house. Three patients, however, reported severe restrictions in their daily activities (13.1%). Patients that were capable of doing sports or heavy labor experienced limited restrictions. However, in the latter two situations, there were six and seven patients, respectively, that could not perform these activities.

**Table 3:** Outcomes measured with the EuraHS quality-of-life scale [33, 34] after complex abdominal wall hernia repair with PADM. Scores are expressed as median scores (range)

Pain at the side of the hernia 0 = no pain, 10 = worst pain imaginable	
Pain in rest (lying down)	0—no pain (range 0–6)
Pain during activities (walking, biking, sports)	0—no pain (range 0–8)
Pain felt during the last week	0—no pain (range 0–10)
Restrictions of activities because of pain or discomfort at the site of the hernia 0 = no restriction, 10 = completely restricted, X = The patient does not perform this activity	
Restriction from daily activities (inside the house)	0 – no restrictions (range 0–10)
Restriction outside the house (walking, biking, driving)	1.5 (range 0–10; 2 times X)
Restriction during sports	1 (range 0–7; 6 times X)
Restriction during heavy labor	2 (range 0–9; 7 times X)
Cosmetic discomfort 0 = very beautiful, 10 = extremely ugly	
Shape of your abdomen	4 (range 0–10)
Site of the hernia	3.5 (range 0–10)

## Discussion

These results show that despite a high rate of postoperative wound infection no biological mesh had to be removed. Both the recurrence rate and the amount of bulging after long-term follow-up are significant (failure rate of 47.8%). The reported quality of life is good after repair of these complex hernias.

The use of biological mesh in complex abdominal wall hernia is relatively new. There are not that many studies comparable with ours regarding methodology and characteristics. The RICH study, in which the results of Strattice™ mesh (Acelity™, non-cross-linked, acellular porcine dermis) were analyzed, seems to have the most similarities in methodological approach [31]. Itani et al. found a recurrence rate of 28% after 2 years [31]. This is comparable with the outcome of this study (26.1%). In the study of Maxwell et al. [43], a much lower recurrence rate of 11.2% was found after median 20.9 months. Patients received an additional CT scan to confirm recurrence. Patients with bulging were excluded from the study. This could lead to certain bias, since bulging is also an unfavorable outcome. In a study by Rosen et al., a much higher recurrence rate of over 50% was found after 3 years of follow-up. In Rosen’s study, the following meshes were assessed: Strattice™ (same mesh; non-cross-linked porcine dermal matrix), Alloderm™ (non-cross-linked human dermal matrix), Biodesign® (porcine small intestinal submucosa sheet), XenMatrix™ (non-cross-linked porcine dermal matrix), and BioA® (biosynthetic web scaffold made of 67% polyglycolic acid (PGA): 33% trimethylene carbonate) [44]. This high recurrence rate could possibly be explained by a longer follow-up. Another difference is the use of a number of different meshes, which could also lead to a higher recurrence rate. Also Itani et al. did not assess the amount of bulging in their study [31]. In this study, a bulging rate of 21.7% was found. This bulging rate is lower compared with data of a previous study from our study group (bulging rate 50.6% [45]). This difference could be explained by the difference in material, cross-linked versus non-cross-linked porcine acellular dermal matrix.

The patients that were enrolled in this study were operated in three hospitals in Germany. Each hospital had at least one surgeon dedicated to abdominal wall surgery. These surgeons treated their patients with PADM. The surgeons also united themselves in the ROKI group to assess their results of PADM repair [32]. Aside from the multicenter character of the study, another important asset was the assessment of quality-of-life parameters. This is only rarely studied in complex abdominal wall hernia repair. These patients suffer not only from a complex abdominal wall hernia but also of multiple comorbidities and risk factors that increase the risk of postoperative complications. A previous study of Roth et al. published about quality-of-life parameters in this specific patient group [46]. In this study, they used the short form-12 health survey [47] and found an improvement of the quality-of-life indicators after 12 months compared with the baseline. This improvement, however, was not significant. In the current study, no comparison was made with the preoperative situation, since these data were not prospectively obtained.

The most frequent postoperative complication in this study was wound infection (39.1%). This percentage is slightly lower than in a recent study of Roth et al. [46]. Roth et al. found 43% wound infections after acellular dermal matrix placement (FlexHD® and Strattice™). The median follow-up was 1 year. The wound infection percentage in our study, however, was somewhat higher than previous studies by Itani el al. [31] (35%), Diaz et al. [48] (33%), Maxwell et al. [43] (26.2%), Helton et al. [49] (23%), and Cheng et al. [50] (5%). All studies [31, 43, 48–50] contained a high percentage of patients that had a hernia classified as clean or clean contaminated [27]. In contrast to our cohort with only contaminated hernias, this could have led to a lower postoperative infection rate. In this study, PADM did not have to be removed representing a better result than found in the studies of Diaz et al. [48] (five mesh removals; 6.7%) and Helton et al. [49] (five mesh removals; 9.8%).

There is contrasting evidence available regarding biological meshes and their characteristics. A recent review by Sainfort et al. [51] assessed the literature about biologic abdominal wall matrices. They concluded that there were no high-level evidence data on biologic prostheses that allowed prioritization of the various biologic prostheses according to their characteristics or their different manufacturing processes. A more recent study by Tripolli et al. [52] assessed the literature on Permacol™, Strattice™, Surgisis®, Tutomesh™, and XenMatrix™. Eleven studies of a poor methodological quality were assessed and included in the review. They concluded that there was a striking statistical variability in the outcomes of all meshes. The only significant finding in their study was that cross-linked meshes had a significantly lower recurrence rate at 12 months than non-cross-linked meshes.

Incisional hernia repair is associated with overall financial losses [53]. Especially biological meshes are expensive and rarely used [54]. In the current study, no cost analysis was performed. In a study of Huntington et al., a cost analysis was performed comparing AlloDerm®, AlloMax™, FlexHD®, Strattice™, and XenMatrix™ [55]. In that study, Strattice™ was the second most expensive mesh (mesh charge per patient US$ 31,875 ± 17,960 and total costs hospital stay per patient US$ 140,394 ± 80,709). The mesh charges and total costs of hospital stay per patient seem higher in the USA than they are in the The Netherlands and Germany; however, these data are illustrative for the current ratios. In another study by Byrge et al., a head-to-head comparison was performed between Permacol™ and Strattice™ meshes in a similar patient group. The costs of the mesh were significantly higher for Strattice™ (median cost $8940) compared with Permacol™ (median costs $1600). The use of Permacol™ resulted in a savings of $181,320 with similar clinical outcomes when compared with Strattice™ [56].

### Limitations

The mesh in this study (PADM) is not used on a large scale, and therefore there is only a limited number of patients that can be assessed. As only patients with potentially contaminated and contaminated abdominal wall hernia were evaluated, there was only a relatively small patient number to include in this study. Although there were more hospitals that work with PADM, not all surgeons seemed that keen to share their data. Another limitation is the design of the study (a cross-sectional cohort study). Data were partly retrospective and partly prospectively collected. This could have led to a bias.

## Conclusion

The data of this study show that despite a high rate of postoperative wound infection, no biological mesh had to be removed. There was a high failure rate of 47.8% due to recurrences and bulging. However, patients reported a good quality of life after repair of these complex hernias without relevant limitation in everyday life activities.
